# X-ray structures of *Na*-GST-1 and *Na*-GST-2 two glutathione s-transferase from the human hookworm *Necator americanus*

**DOI:** 10.1186/1472-6807-7-42

**Published:** 2007-06-26

**Authors:** Oluwatoyin A Asojo, Kohei Homma, Meghan Sedlacek, Michelle Ngamelue, Gaddam N Goud, Bin Zhan, Vehid Deumic, Oluyomi Asojo, Peter J Hotez

**Affiliations:** 1 Department of Pathology and Microbiology, College of Medicine Nebraska Medical Center, Omaha NE 68198-6495, USA; 2 Department of Microbiology, Immunology, and Tropical Medicine, The George Washington University Medical Center, Washington DC, 20037, USA

## Abstract

**Background:**

Human hookworm infection is a major cause of anemia and malnutrition of adults and children in the developing world. As part of on-going efforts to control hookworm infection, The Human Hookworm Vaccine Initiative has identified candidate vaccine antigens from the infective L3 larval stages and adult stages of the parasite. Adult stage antigens include the cytosolic glutathione-S-transferases (GSTs). Nematode GSTs facilitate the inactivation and degradation of a variety of electrophilic substrates (drugs) via the nucleophilic addition of reduced glutathione. Parasite GSTs also play significant roles in multi-drug resistance and the modulation of host-immune defense mechanisms.

**Results:**

The crystal structures of *Na*-GST-1 and *Na*-GST-2, two major GSTs from *Necator americanus *the main human hookworm parasite, have been solved at the resolution limits of 2.4 Å and 1.9 Å respectively. The structure of *Na*-GST-1 was refined to R-factor 18.9% (R-free 28.3%) while that of *Na*-GST-2 was refined to R-factor 17.1% (R-free 21.7%). Glutathione usurped during the fermentation process in bound in the glutathione binding site (G-site) of each monomer of *Na*-GST-2. *Na*-GST-1 is uncomplexed and its G-site is abrogated by Gln 50. These first structures of human hookworm parasite GSTs could aid the design of novel hookworm drugs.

**Conclusion:**

The 3-dimensional structures of *Na*-GST-1 and *Na*-GST-2 show two views of human hookworm GSTs. While the GST-complex structure of *Na*-GST-2 reveals a typical GST G-site that of *Na*-GST-1 suggests that there is some conformational flexibility required in order to bind the substrate GST. In addition, the overall binding cavities for both are larger, more open, as well as more accessible to diverse ligands than those of GSTs from organisms that have other major detoxifying mechanisms. The results from this study could aid in the design of novel drugs and vaccine antigens.

## Background

In 1962, Dr. Norman Stoll of the Rockefeller foundation referred to human hookworm infection ('hookworm') as the "The Great Infection of Mankind" and today, almost half a century later, this remains the case [1]. Hookworm is still highly endemic globally, with approximately 576 million cases [2]. *Necator americanus *is considered the most common hookworm worldwide. In the developing regions of sub-Saharan Africa, Asia, and the Americas, hookworm is an important cause of iron-deficiency anemia and protein malnutrition; it is considered the second most important parasitic disease next to malaria [1]. Both of these pathologic processes result from the blood-feeding activities of adult hookworms in the host small intestine. It is estimated that approximately 25 adult *N. americanus *hookworms cause the loss of approximately 1 ml of blood daily [3]. Two populations are particularly vulnerable to developing hookworm anemia – children and women of reproductive age, including pregnant women [4]. In children, chronic hookworm anemia results in impaired growth and cognitive development [4], and a recent economic analysis suggests that these processes result in significant wage earning losses [5]. In pregnant women, hookworm is a major contributor to severe anemia in pregnancy, low birth weight, and increased maternal morbidity and mortality [6].

In response to the increasing awareness of hookworm as a major public health threat, the World Health Organization and other international agencies currently advocate global efforts to control hookworm morbidity through the regular and periodic use of anthelmintics [7]. In areas of high hookworm transmission it is recommended to administer anthelmintics, usually a single dose of either albendazole or mebendazole, two-three times annually. Although this 'deworming' approach would lead to important morbidity reduction, there are larger concerns about its sustainability given 1) the high rates of hookworm re-infection following drug treatment [8], 2) the diminishing efficacy of the drug with repeated use [9], possibly because of drug resistance [10], and 3) the high prevalence and intensity of hookworm infection among adult populations, most notably women of reproductive age [11].

In response to these concerns, an international product development partnership known as The Human Hookworm Vaccine Initiative [12] was initiated to develop an anti-hookworm vaccine aimed at reducing worm burdens and intensity, as an alternative or complementary approach to deworming [1,13,14]. The HHVI has identified promising vaccine candidates from both the adult stages of hookworms, as well as the infective larval stages [13,15]. Ultimately, the HHVI is working to develop multivalent vaccine comprised of at least one larval and one adult antigen. The larval antigen, ASP-2, pdb-code 1U53 [16], has undergone pilot scale manufacture, and Phase 1 clinical testing [13], while two adult antigens, including an aspartic protease (APR-1) [17] and a glutathione-S-transferase (GST) [18] are at earlier stages of development.

The GST superfamily is comprised of widely distributed isoenzymes that perform functions as diverse as the detoxification of electrophilic compounds to protecting against peroxidative damage [19]. GSTs are homodimers that catalyze the nucleophilic addition of reduced glutathione to various diverse electrophilic substrates consequently facilitating their inactivation, and extrusion [20,21]. GSTs are a major detoxification system for helminths, which have limited detoxification enzymes and apparently lack the cytochrome P-450 dependent reactions [22-24]. Thus, GSTs are critical to the survival of adult helminths in the host. Inhibition of GSTs will deprive parasitic helminths of their major detoxification and defense against oxidative stress, making hookworm GSTs a viable target for the design of novel vaccines as well as anthelmintics. A 28 kDa GST from *Schistosoma haematobium*, the etiologic agent of urinary schistosomiasis is undergoing clinical trials in Africa [25].

In preclinical studies conducted in laboratory dogs and hamsters challenged with infective hookworm larvae, Ac-GST-1, a GST from the canine hookworm, *Ancylosotma caninum *demonstrated promise as vaccines that reduce adult worm burden and fecundity of female worms [18]. Based on these laboratory animal vaccine trials, enzymatic and structural studies were initiated recently to provide new insights into the roles of hookworm GSTs as functional vaccines. In order to clarify the role of hookworm GSTs as possible drug and vaccine targets, we have undertaken X-ray structural analysis of the *Na*-GST-1 and *Na*-GST-2, two of the three known GSTs from *N. americanus*. We present here first structures of human hookworm GSTs.

## Results and discussion

### Crystallization and structure determination

The structures of two major GSTs, *Na*-GST-1 and *Na*-GST-2 from *Necator americanus*, have been determined using recombinant protein expressed in and secreted by *Pichia pastoris*. Both *Na*-GST-1 and *Na*-GST-2 crystals were obtained from solutions containing a high percentage of polyethylene glycol. The best crystals were obtained by vapor diffusion in sitting drop comprised of equal volumes of 18 mg/ml protein and precipitant solution. Precipitant solutions were comprised of 0.1 M sodium acetate pH 4.6 and 30% PEG 400 for *Na*-GST-1. The precipitant solution for *Na*-GST-2 was 18% PEG4000, 0.1125 M HEPES pH 7.55, 11.25% isopropanol, 0.01 M sodium acetate and 0.06 M sodium citrate, to yield a final pH of 7.25. The statistics for data collection and refinement are shown in Table 1.

**Table 1:** Data Collection and model refinement statistics

Data Collection	*Na*-GST-1	*Na*-GST-2
Space group	*P*2_1_2_1_2_1_	P2_1_
Resolution (Å)	30–2.4 (2.5–2.4)	30–1.9 (2.0–1.9)
R_merge _(%)^a^	6.0 (19.1)	6.0 (36.0)
Completeness (%)	97.7 (88.1)	88.0 (69.8)
Redundancy	5.3 (3.2)	2.9 (2.1)
I/σ(I)	11.2 (4.5)	12.1 (2.5)
Refinement		
R-factor^b ^(%)	18.9 (17.4)	17.6 (21.8)
R-free^c ^(%)	28.3 (33.1)	21.7 (30.2)
Correlation coefficient		
Fo – Fc	0.902	0.958
Fo – Fc Free	0.945	0.936
R.m.s. deviation		
bond length (Å)	0.017	0.014
bond angles (°)	1.675	1.383
Ramachandran plot		
Most preferred (%)	91.0	93.7
Additional allowed (%)	7.8	5.7
Generously allowed (%)	0.8	0.6
Disallowed (%)	0.4	0.0
Model Composition asymmetric unit		
Number of monomers	4	8
Number of glutathione bound	0	8

^a ^Rmerge = (Σ|I - <I>|)/ΣI, Where I is the observed intensity, and <I> is the average intensity obtained from multiple observations of symmetry-related reflections after rejections.

^b^R-factor = Σ||Fo| - |Fc||/Σ|Fo| where Fo are observed and Fc are calculated structure factors amplitudes.

^c ^R-free set uses 5% of randomly chosen reflections.

Initial phases were obtained by molecular replacement (MR) using the program PHASER [26-31] with a polyalanine model based on a monomer of HpolGST from the nematode *H. polygyrus *(pdb code 1TW9) as the search model [32]. The cell constants and space groups were a = 50Å, b = 81Å, c = 201Å, P2_1_2_1_2_1 _for *Na*-GST-1 and a = 58Å, b = 108Å, c = 167Å β = 90.02°, P2_1 _for *Na*-GST-2. The orthorhombic *Na*-GST-1 contains a tetramer per asymmetric unit which corresponds to a Matthews' coefficient of 2.3 Å/Da and solvent content of 46%. Monoclinic *Na*-GST-2 contains an octamer in the asymmetric unit, corresponding to a Matthews' coefficient of 2.8 Å/Da and solvent content of 55%.

Although the data set for *Na*-GST-2 could be processed as orthorhombic and a molecular replacement solution could be obtained in the space group P222_1 _having 4 monomers in the asymmetric unit, refinement stalled at the unreasonably high R-factor of 50% (R-free 55%). In an effort to confirm the space group, we generated a new search model from a monomer of the structure of *Na*-GST-2 that had been refined in the monoclinic space group. Using this search model, we repeated PHASER in the automatic mode with each of the alternative primitive orthorhombic space groups. A molecular replacement solution with reasonable packing was obtained in P222_1 _having 4 monomers in the asymmetric unit, and the same rotation and translational function as with the polyalanine search model. However, despite having 2Fo-Fc maps that agreed well with the model, both the R-factors and free R-factors remained above 48% even after extensive positional refinement with CNS, and/or REFMAC, taking advantage of extensive non crystallographic symmetry (NCS). Simulated annealing with CNS in either carthesian or torsional mode at the following temperatures 2500 K, 5000 K, or even 10,000 K did not reduce the free R-factor while the R-factor did not drop below 35%. The inability to refine the model in the orthorhombic space group, suggests that the space group is indeed monoclinic P2_1_. Furthermore, no other orthorhombic space groups yielded a molecular replacement solution with reasonable packing and no overlaps.

**Figure 1:**
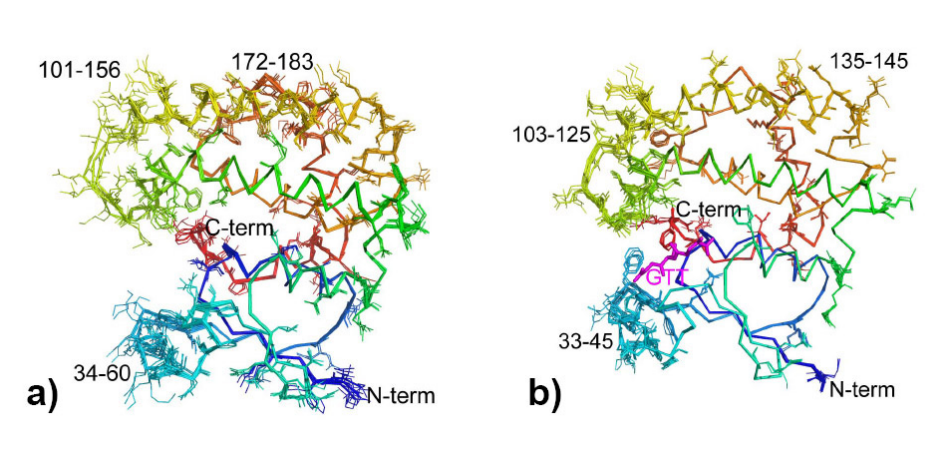
Structural alignment of the a) four *Na*-GST-1 molecules and b) eight *Na*-GST-2 molecules in the crystallographic asymmetric unit, colored in rainbow from blue (N-termini) to red (C-termini). Side-chains in the regions of greatest variation are shown as sticks.

The final refined monoclinic P2_1 _model of *Na*-GST-2 indicates that differences in subunit structures prevent the higher order symmetry, and there are differences in side chain orientation across the monomers, and main chain residues; both termini; G-site vicinity (33–45); as well as in proximity to the dimerization domain (residues 101–156, 172–183) which are capable of breaking the higher order symmetry (Fig. 1). Overall the monomers of *Na*-GST-2 align better than those of *Na*-GST-1 with r.m.s. deviation of 0.16 Å versus 0.38 Å for all main chain atoms. In both structures the largest variation is observed in the regions bordering the G-site and anchoring the dimerization domain (Fig. 1). These regions were previously observed to be the regions of highest variation in the only other known hookworm GST structure, that of HpolGST from the nematode *H. polygyrus *(pdb code 1TW9) [32].

The final models for both *Na*-GST-2 and *Na*-GST-1 structures were obtained following iterative cycles of model building in O [33] and structure refinement in REFMAC-5 with NCS averaging [34-36]. *Na*-GST-1 was refined to R-factor 18.9% (R-free 28.3%) at 2.4 Å, while *Na*-GST-2 was refined to R-factor 17.1% (R-free 21.7%) at 1.9 Å.

All the main chain atoms for all 206 amino acids for all monomers were ordered in the *Na*-GST-1 structures however some monomers have disordered side chain residues, most prominently in the region from amino acids 105 through 132, interestingly these residues are include some of those that break the higher order symmetry of the *Na*-GST-2 structure. In the *Na*-GST-2 structure, all main chain and side chain atoms for each monomer were visible in 2Fo-Fc omit maps calculated from the molecular replacement solutions.

The main chain and side chain stereochemistry of the refined models are excellent and 98% of main chain phi/psi angles lie within the allowed region of a Ramachandran plot. More details of the quality of the structure as well as data collection are shown in Table 1. The atomic coordinates and structure factors have been deposited in the PDB under accession numbers 2ON5 and 2ON7 for *Na*-GST-2 and *Na*-GST-1 respectively.

### Structural features

**Figure 2:**
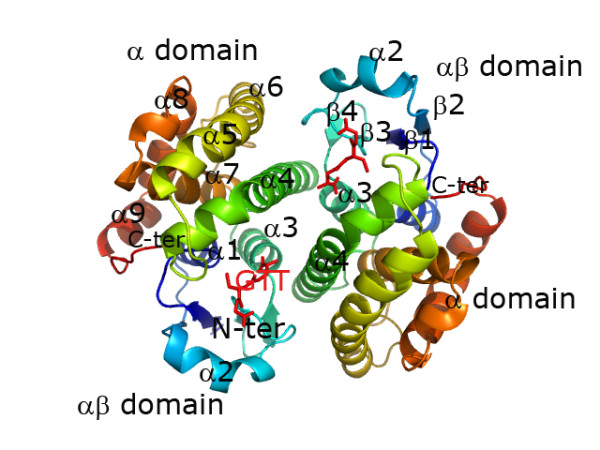
Ribbon representation of *Na*-GST-2 dimer reveals a typical GST dimer.

The final refined model of *Na*-GST-1 has four monomers in the asymmetric unit, while *Na*-GST-2 has eight monomers in the asymmetric unit. As is the case with all GSTs, both *Na*-GST-1 and *Na*-GST-2 form dimers. The fundamental/active units of both structures are homodimers with the classical GST topology of Nu class nematode specific GSTs (Fig 2). Each monomer of the homodimer is related by a 2-fold axis of symmetry. The highly conserved N-terminal glutathione binding site is embedded within an alpha-beta domain while the more variable C-terminal ligand binding site is in the helical alpha domain (Fig. 2).

**Figure 3:**
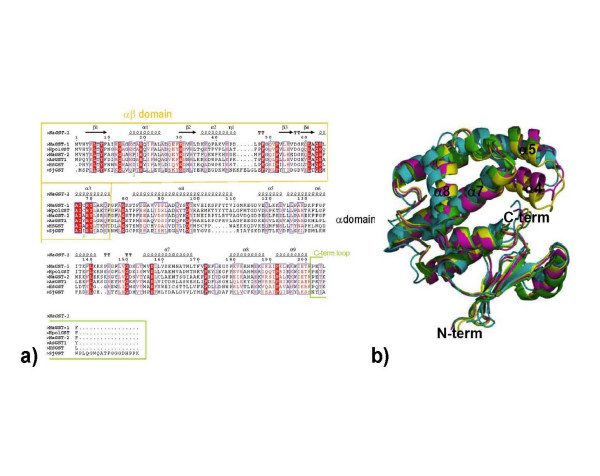
Sequence and structural alignment of Nu class GSTs with a Sigma Class GST (HsGST, human GST or hematopoietic prostagladin D synthase [37]) and other parasite GSTs (SjGST, *Schistosoma japonica* [43], *Ascaris suum *(*As*-GST-1) [42]. (a) The alignment reveals that firstly N-terminal alpha beta domain is more conserved than the C-terminal alpha domain. Furthermore, *Na*-GST-1 has higher sequence identity with HpolGST than *Na*-GST-2 and the lowest similarity is with the HsGST. This figure was generated with ESPript [55, 56]. (b) Structural alignment of monomers of Nu class GSTs (*Na*-GST-1, magenta; *Na*-GST-2, gold; HpolGST, green) with a sigma class GST (HsGST, cyan).

**Figure 4:**
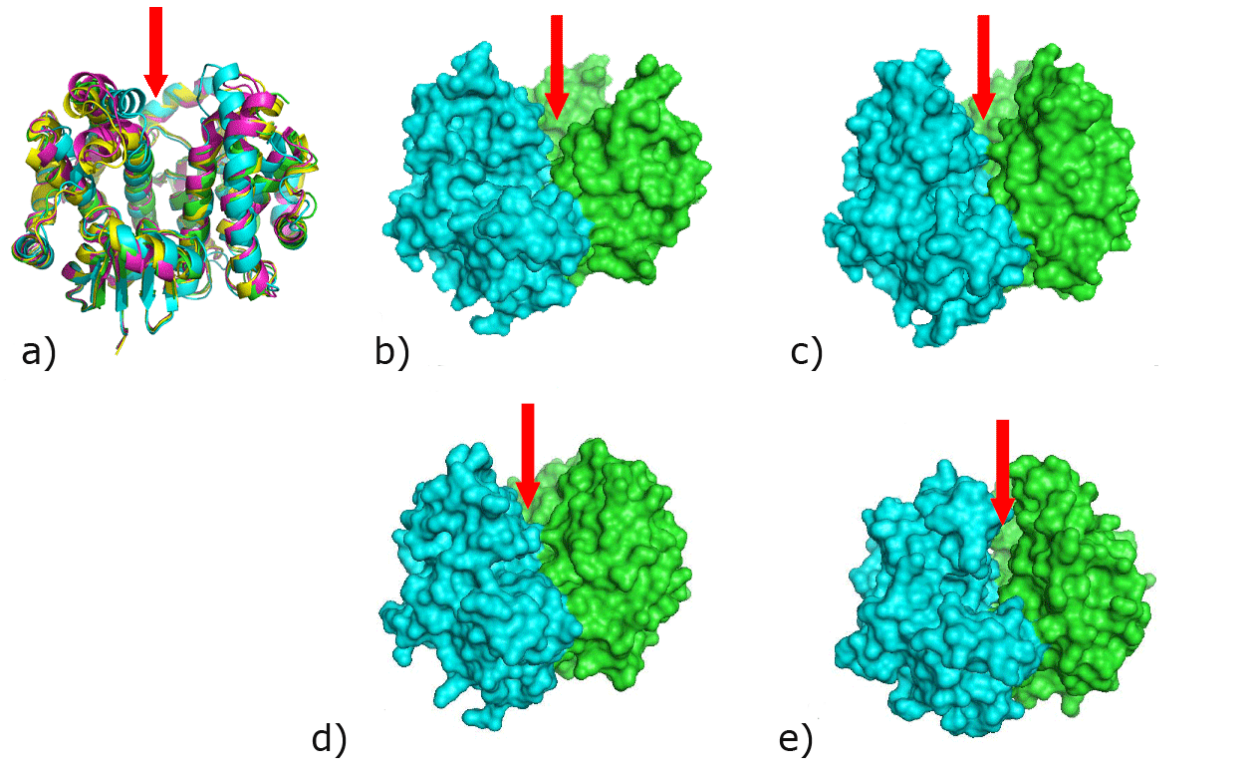
Comparison of GST dimers. a) Superposition of GST dimers reveals that they are very similar, however, Nu class (*Na*-GST-1, magenta; *Na*-GST-2, gold; HpolGST, green) have a more accessible binding cavity than sigma class (HsGST, cyan). The path to the binding cavity is indicated by the red arrow. The surface plots of Nu class GSTs b) HpolGST c) *Na*-GST-1 d) *Na*-GST-2 reveal larger access way to binding cavity than e) sigma class GST (HsGST).

The primary and tertiary structures of *Na*-GST-1 are more like that of HpolGST than *Na*-GST-2 (Fig. 3). *Na*-GST-1 has higher sequence similarity with HpolGST than *Na*-GST-2 67% versus 61% the same goes for r.m.s deviation 0.84 Å versus 0.91 Å, for all main chain atoms. However, the monomers and dimers are also quite similar to that of human GST such as represented by hematopoietic prostagladin D synthase (HsGST) [37] (Fig. 3. & Fig. 4). *Na*-GST-1 has lower sequence similarity with HsGST than *Na*-GST-2 34% versus 38% but higher r.m.s deviation for alignment of dimers is 1.592 Å versus 1.434 Å, for all main chain atoms. Interestingly the regions of highest variability are along the dimer interface, most notably from helices α4 and α5 as well as the relative orientation of helix α8 (Fig. 4). The variation in these regions leads to a considerably difference in the size of binding cavity and accessibility to said cavity of the GSTs (Fig. 4). Large portions of the loop linking helices α4 and α5 are disordered in the HpolGST structure. The structures reveal that in the Nu class nematode specific GSTs the monomers form a more open embrace around the binding cavity, as compared to human GST, resulting in wider cavities that are more accessible to larger compounds. This is inline with the role of these GSTs as the major detoxification mechanism for the hookworm parasite. Furthermore, the cavity is more open in the absence of glutathione (in the case of *Na*-GST-1) than with glutathione bound (in the case of *Na*-GST-2), suggesting a conformational variation upon glutathione binding.

### G-site features

**Figure 5:**
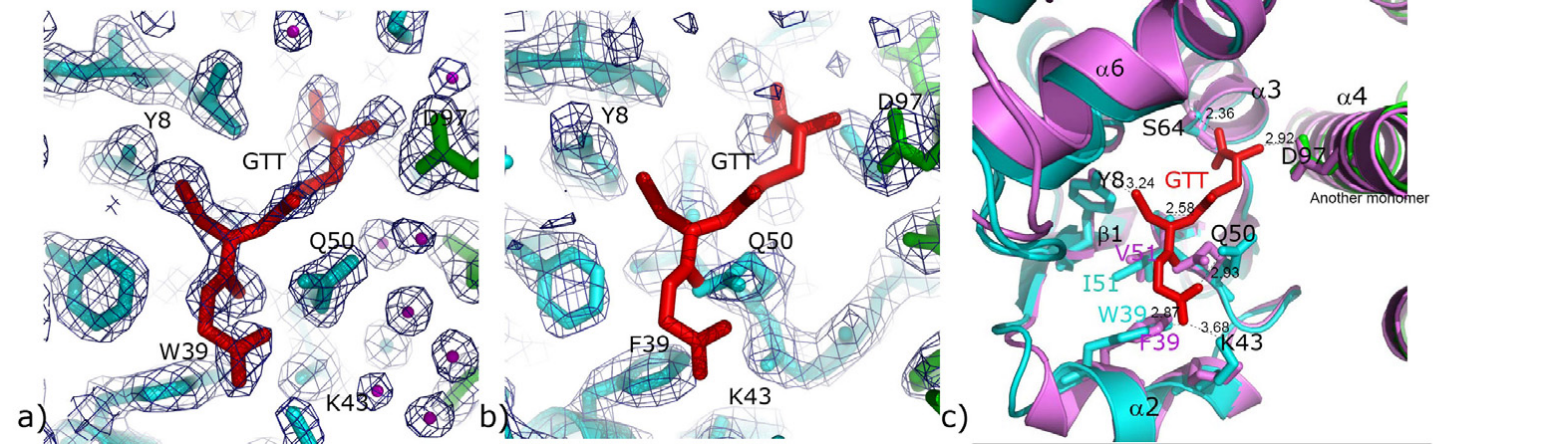
G-site features. a) G-site of *Na*-GST-2 shows unambiguous electron density for glutathione (GTT) in 2Fo-Fc maps contoured at 1 sigma. b) No such density is visible in the G-site of *Na*-GST-1. c) Alignment of G-sites of *Na*-GST-2 and *Na*-GST-1. GTT is modeled from *Na*-GST-2 structure. The monomers of *Na*-GST-2 dimer are colored in cyan and green, while *Na*-GST-1 is colored in violet. Trp39 forms a hydrogen bond with glutathione in *Na*-GST-2 which is replaced with Phe39 in *Na*-GST-1. Gln50 is conserved in both *Na*-GST-2 and *Na*-GST-1, but the side chain is flipped such that glutathione cannot fit in *Na*-GST-1 G-site. The catalytic Tyr8 maintains its conformation in both structures. Polar interactions and distances are also shown.

Although no glutathione was added to the crystallization mixture, unambiguous density for a glutathione molecule was observed in G-site of each monomer of *Na*-GST-2 (Fig. 5). Apparently, *Na*-GST-2 usurps glutathione during the fermentation process. No such density was observed in the electron density maps for *Na*-GST-1, instead scattered density were visible in the substrate binding cavity (Fig. 5). Interestingly, the G-site of *Na*-GST-1 cannot fit glutathione as it is abrogated by Gln 50 which points directly in the binding cavity blocking glutathione binding. A rotation of Gln around the Cβ will allow sufficient room for gluthatione to bind. It is plausible that a pH change may facilitate this rotation. *Na*-GST-1 was crystallized at an acidic pH 4.6 whereas *Na*-GST-2 was crystallized at a more basic pH of 7.5. Evidently, *Na*-GST-1 is capable of binding glutathione and like *Na*-GST-2 is catalytically active at pH 6.8 as measured by glutathione conjugation with 1-chloro-2, 4-dinitrobenzene (CDNB) [38,39].

As was observed in other GSTs, the conserved, catalytic Tyr (Tyr 8) stabilizes the Cys moiety of glutathione. Tyr 8 forms a hydrogen bond with the sulfur of glutathione. The formation of this hydrogen bond interaction may result in a lower pKa for the thiol in the GST-glutathione complex [40,41]. The main chain oxygen and nitrogen of Ile 51 form hydrogen bonds with the nitrogen and oxygen of the Cys of glutathione (Fig. 5). The side chain glutamyl residues of glutathione face the inter-domain cleft and are stabilized by hydrogen bonds with Trp 39 (Fig. 5). Trp 39 is conserved in sigma class of GSTs, typified by hematopoietic prostagladin D synthase [37] whereas *Na*-GST-1 like another Nu class GST HpolGST has a Phe in that position. Phe 39 is incapable of forming the same type of hydrogen bond however Lys 43 is in close enough proximity to form the same interaction as Trp 39. In addition, the glycyl residue in glutathione forms hydrogen bonds with Ser 64, while having an inter-molecular hydrogen bond from the conserved Asp 97 from across the dimer interface (Fig. 5). Our *Na*-GST-2 structure gives the first view of glutathione binding in a Nu class GST [32].

### H-site features for Nu class GSTs

**Figure 6:**
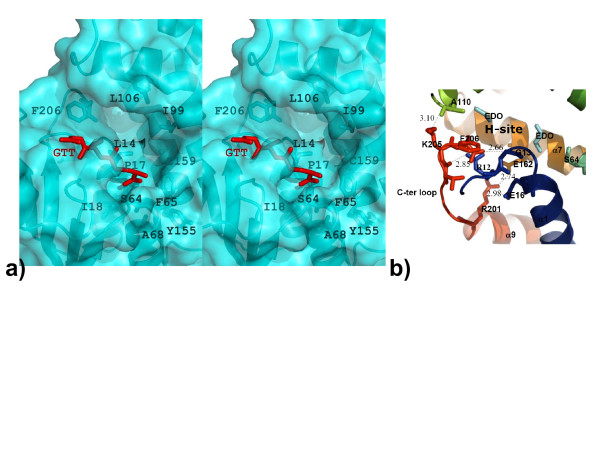
H-site features. a) Stereo representation of the H-site of *Na*-GST-2, residues that form the cleft are shown as sticks. b) The C-terminal loop of *Na*-GST-2 is stabilized by a network of hydrogen bonds, residues involved in these interactions are shown in as sticks, while the bonds are indicated by dashed lines and distances are shown. Residues are colored in rainbow representation from N-terminal to C-terminal (Blue-Green-Yellow-Orange-Red). Two ethylene glycol molecules (EDO, in cyan) from the cryo-protecting liquor are visible in the surface of the H-site.

**Figure 7:**
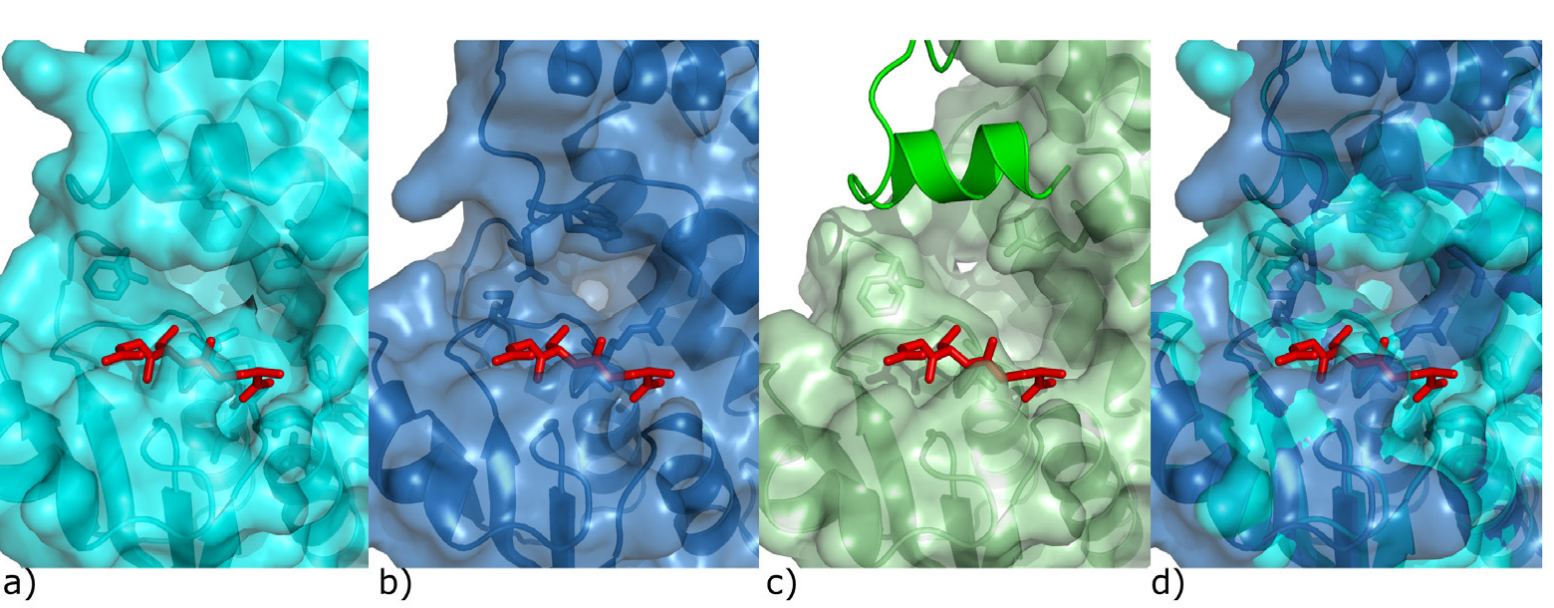
Nu class GSTs {a) *Na*-GST-2, c) HpolGST} have larger binding cavity than sigma class GST {b) HsGST}. d) Overlay of the cavities reveals the considerable reduction in the active site size between sigma class (blue) and nu class (cyan). The structure of HpolGST is missing a loop in close proximity to the binding cavity and we modeled it as cartoon from the *Na*-GST-2 structure. The glutathione in the G-site is shown as red stick model.

Ligand binding or H-sites structures vary across classes of GSTs because the C-terminal H-sites are largely responsible for the varying substrate specificities of the GSTs. As was observed in the structure of HpolGST, the H-site of Nu class GSTs as represented by *Na*-GST-2 forms a long, deep cleft (Fig. 6). This cleft is formed by the interaction between hydrophobic residues Gly 13, Ala/Leu 14, Leu/Phe 65 from the α3 domain with the residues Tyr 95, Phe/Tyr 106 and Phe 206 from the α/β domain. There is an additional stabilizing salt bridge from Glu 162 to Arg 201 (Fig 6). In the *Na*-GST-2 structure there are some ethylene glycol molecules from the cryoprotectant solution lining the surface of the H-site (Fig. 6). The significance of the ethylene glycol molecules remains unclear until we probe the H-site with a suitable ligand. The primary and tertiary structures of the H-sites of the Nu class GSTs overlay quite well (Fig. 3). *Na*-GST-1 and *Na*-GST-2 as was observed for HpolGST [32] exhibit H-sites or ligand binding sites that are larger and more receptive to longer, larger inhibitors which cannot fit in the smaller H-site of mammalian GST (Fig. 7). Overlaying of the cavities reveals the considerable reduction in the active site size between sigma class and nu class (Fig. 7). This, in addition to the more open and accessible binding cavity (Fig. 4) suggests that it is possible for the GSTs to play the role of the major detoxifying system in the hookworm parasite.

### Drug inhibition and implications for drug design

We examined if conventional anthelmintics were capable of competitively inhibiting the hookworm GSTs by measuring the conjugation of CDNB with glutathione. We then compared our activity data to that of model roundworm *Ascaris suum *(*As*-GST-1) which used the same assay and conditions as we did [42]. Like hookworms, tapeworms are treated with albendazole or mebendazole. Our results show that albendazole has approximately 5 times lower IC50 for *Na*-GST-1 and 7 times lower IC50 for *Na*-GST-2 than *As*-GST-1, Table 2. The crystal structure of As-GST-1 has not been solved but comparing the primary sequence of *As*-GST-1 with the Nu class GSTs reveals a high sequence similarity even in the C-terminus alpha domain (Fig. 3a), suggesting similar substrate specificity and ligand binding. However, *As*-GST-1 some large residues which may obscure the H-site (Lys 183, Lys 203 and Trp 167), accounting for the higher IC50.

**Table 2:** Estimated inhibition constants of GSTs by conventional anthelmintics

Enzyme	*Na*-GST-1	*Na*-GST-2	*As*-GST-1*
Inhibitor	IC_50 _(μM)	IC_50 _(μM)	IC_50 _(μM)
Albendazole	87	68	520.0
Chlorotriphenyltin	22	0.7	0.3
Praziquantel	(>20,000)**	3300	--

* Data from *As*-GST-1 is from reference [42]

** This was the maximum concentration tried due to solubility issues.

The IC50 for both albendazole and chlorotriphenyltin were higher for *Na*-GST-1 than *Na*-GST-2 (Table 2). Chlorotriphenyltin was the most effective inhibitor of *Na*-GST-1, *Na*-GST-2 as well as As-GST-1. This is not surprising based on the relatively small size of chlorotriphenyltin compared to albendazole. Chlorotriphenyltin successfully competes with CDNB for glutathione conjugation as it is more likely to have access to the H-site, than albendazole, however it is doubtful that chlorotriphenyltin is specific for any class of GSTs. In order to clarify the mode of drug binding, we are currently attempting to co-crystallize both hookworm GSTs with albendazole or chlorotriphenyltin.

Neither *Na*-GST-1 and *Na*-GST-2 are effectively inhibited by praziquantel, however, praziquantel is a broad spectrum anthelmintic and is the leading drug for the treatment of *schistosomiasis*. The structure of the *Sj*GST from *Schistosoma japonica *in complex with praziquantel has been solved and in this structure, praziquantel appears to bind in the G-site [43]. It was also observed that praziquantel does not competitively inhibit recombinant *Sj*GST [44], suggesting that *Sj*GST is not the target of praziquantel as was speculated by McTigue and colleagues [43]. In fact based on our data we may conclude that neither *Na*-GST-1 nor *Na*-GST-2 is targeted by praziquantel.

## Conclusion

The 3-dimensional structures of *Na*-GST-1 and *Na*-GST-2 are presented here. The GST-complex structure of *Na*-GST-2 reveals a typical GST, while G-site that of *Na*-GST-1 is abrogated by Gln 50 which suggests that some conformational flexibility is required in order to bind the substrate GST. The overall binding cavities for both are larger, more open and appear to be more accessible to diverse ligands than those of GSTs from organisms that have other major detoxifying mechanisms. Ongoing structural studies with larger ligands are underway.

## Methods

### Molecular cloning and expression

A cDNA library of of *N. americanus *infective larvae (L3) was constructed as previously described [45,46], cDNA encoding *Na*-GST-2 and *Na*-GST-1 were isolated by immunoscreening of *N. americanus *L3 cDNA expression library using antiserum against *Ac*-GST-1 from *A. caninium *followed by cDNA cloning and sequencing [45]. The entire coding sequence was PCR amplified from the first strand cDNA of adult *N. americanus *with gene-specific primers. The PCR products were sub-cloned into the *Pichia *expression vector pPICZαA (Invitrogen) via the XhoI and XbaI sites. The correct insert and right reading frame were confirmed by double strand sequencing of recombinant plasmid using flanking vector primer: α-factor and 3' AOX1. Fermentation and large scale purification were carried out according to the protocols described for *Na-ASP-2 *[47] and elsewhere (Goud et al. to be published).

### Crystallization and data collection

All crystals were grown at 22°C by vapor diffusion in sitting drops. Both *Na*-GST-1 and *Na*-GST-2 was concentrated to 18 mg/mL in 100 mM Tris HCL pH 8.0, prior to screening for crystallization conditions. Initial crystallization screens were performed using the following commercial screens from Nextal Biotechnologies (Qiagen) Classics, PEG and Cryo. Single chunk-like *Na*-GST-1 crystals were obtained from 0.1 M sodium acetate pH 4.6 and 30% PEG 400 (or 25% PEG MME 500). Initial *Na*-GST-2 crystals were flat, thin stacked plates which were smaller than 0.03 mm on the smallest face. Following further optimization using the Nextal OptiSalt pre-filled screens, thicker rods were obtained in 18% PEG 4000, 0.1125 M HEPES pH 7.55, 11.25% isopropanol, 0.01 M sodium acetate pH 4.6, 0.06 M sodium citrate. *Na*-GST-1 crystals were cryo-protected by adding 15% trehalose directly to the drop. *Na*-GST-2 crystals were transferred to a cryo-protecting solution comprised of the precipitants and 20% ethylene glycol prior to flash cooling. Crystals were flash cooled in a stream of N2 (X-stream 2000 low temperature system, RigakuMSC) prior to data collection. The X-ray system consisted of RuH3R rotating anode generator (RigakuMSC) operating at 50 kV and 100 mA, with Osmic Micromax optics and an R-axis IV++ image plate detector (RigakuMSC). Data were collected from a single crystal using a crystal-to-detector distance of 200 mm and exposure times of 20 minutes for 0.5° oscillations.

All X-ray data sets were collected using Crystal Clear (d*trek) package [48]. Data was processed using MosFLM [49,50]. The structures were solved by molecular replacement with PHASER [26-31]. This was followed by iterative cycles of manual model building with the program O [33] and structure refinement with REFMAC5 [35,36] using a maximum likelihood refinement procedure with Engh & Huber Geometric parameters and free-R [51]. Data and refined model statistics are shown in Table 1. The superposition of models and r.m.s. deviations were calculated using the program Swiss-PdbViewer Version 3.7 [52,53]. Unless otherwise noted, figures were generated using pyMOL [54].

### Activity studies

GST activity and drug inhibition was determined in a standard assay measuring the conjugation of 1-chloro-2, 4-dinitrobenzene (CDNB) with glutathione [38,39]. The assay mixture contained in a total of 1 mL, 1 mM CDND, 1 mM glutathione and 0.1 M potassium phosphate buffer, pH 6.5. To determine GST activity, the rate of increase of absorbance at 340 nm, were taken with a NanoDrop ND-1000 Spectrophotometer every thirty seconds for 3 minutes upon initiation of the reaction by the addition of varying concentrations of each enzyme.

Drug inhibition was measured using a fixed concentration of enzyme (1 μM) and using concentration, from 1 nM to 20 mM, of the following compounds 1) albendazole (MP Biomedicals), 2) praziquantel (Alexis) and 3) chlorotriphenyltin (Acros Organics) to inhibit in the glutathione CNDB conjugation reaction. Water insoluble compounds were dissolved in 100% ethanol. Absorbance readings, at 340 nm, were taken with a NanoDrop ND-1000 Spectrophotometer every thirty seconds for 3 minutes upon initiation of the reaction by the addition of 1 μM enzyme. The 50% enzymatic inhibitory concentrations (IC50) were determined from direct plots of percent activity against inhibitor concentration from data points generated from at least 4 repetitions of each time point and concentration.

## Authors' contributions

OAA and PJH conceived the project; OAA grew crystals, solved, refined the crystal structures, generated figures and composed the manuscript; KH, analyzed for drug binding, collected, generated figures and processed all the data sets; MS planned and performed all activity studies; GNG and VD fermented and purified the protein samples; BZ cloned *Na*-GST-1 and *Na*-GST-2 cDNA; MS, MN, OAA and OA grew crystals, screened for and soaked crystals with drugs. All authors have approved the final manuscript.

## Supplementary Material

Additional file 1 Pre-release coordinates of the structure of *Na*-GST-1. Click here for file

Additional file 2 Pre-release coordinates of the structure of *Na*-GST-2. Click here for file
